# Transcriptome and Metabolome Profiling of a Novel Isolate *Chlorella sorokiniana* G32 (Chlorophyta) Displaying Enhanced Starch Accumulation at High Growth Rate Under Mixotrophic Condition

**DOI:** 10.3389/fmicb.2021.760307

**Published:** 2022-01-06

**Authors:** Qingling Zhu, Mengmeng Zhang, Bingying Liu, Fang Wen, Zhili Yang, Jianhua Liu

**Affiliations:** ^1^Systems Biology, School of Marine Science and Technology, Zhejiang Ocean University, Zhoushan, China; ^2^Marine Biology, Ocean College, Zhejiang University, Zhoushan, China; ^3^National Engineering Research Center for Marine Aquaculture, Zhejiang Ocean University, Zhoushan, China

**Keywords:** *Chlorella sorokiniana*, glucose-responsive genes, metabolome, mixotrophic growth, transcriptome (RNA-seq)

## Abstract

*Chlorella sorokiniana* is one of the most productive microalgal species with a high potential for the production of biofuels and other high value-added molecules. Many studies have focused on its capability of mixotrophic growth using reduced organic carbon and growth pattern shift between autotrophic and mixotrophic conditions. In this study, we investigated growth patterns of a novel isolate, *C. sorokiniana* G32, under mixotrophic growth conditions supplemented with a low level (1.25 g L^–1^) and a high level (5 g L^–1^) of glucose. Physiological, transcriptomic (i.e., RNA-seq), and metabolomic (i.e., LC-MS/MS) methods were used. We showed that peak growth based on OD_680nm_ absorbance is ∼4-fold higher with high glucose vs. low glucose supplementation. Photosynthetic efficiency (Fv/Fm) in G32 mixotrophic cultures with high or low glucose supplementation remains identical to that of G32 phototrophic growth. We also found that the conversion rate between absorbance-based cell density and cell dry weight with high glucose supplementation was lower than with low glucose. This suggests that more cell biomass is produced under high glucose treatment than with low glucose. The result was confirmed via sucrose density gradient centrifugation. It is likely that accumulation of high concentration of starch may account for this effect. Transcriptomic analysis of G32 cultures (i.e., via RNA-seq) in response to reciprocal change of glucose levels reveals that expression of a subset of differentially expressed genes (DEGs) is correlated with the amount of glucose supplementation. These DEGs are designated as glucose-specific responsive (GSR) genes. GSR genes are enriched for a number of energy metabolic pathways. Together with metabolomics data (i.e., LC-MS/MS), we show that under high-level supplementation, glucose is preferentially oxidized through an oxidative pentose phosphate pathway. Collectively, our results indicate the mechanism of regulation of glucose assimilation and energy metabolism in G32 under mixotrophic conditions with different levels of glucose supplementation revealed by transcriptomic and metabolomic analyses. We propose that *C. sorokiniana* G32 has the potential for the production of high value-added molecules.

## Introduction

There is renewed interest in microalgae for their potential in the production of biofuels and other high value-added molecules. Specifically, microalgae are considered to have high photosynthetic efficiency and growth rate compared with other photosynthetic organisms, allowing for rapid, high biomass accumulation (Herrero et al., 2006; Spolaore et al., 2006; Chisti, 2007; Unkefer et al., 2017; Randhir et al., 2020). Some microalgae species such as *Chlorella sorokiniana* are capable of mixotrophic growth using both photosynthetic carbon and supplemented organic carbons from molecules such as glucose, glycerol, ethanol, and acetate (Wan et al., 2011; Li et al., 2014; Cecchin et al., 2018). Other species, such as *Nannochloropsis salina*, exhibit reduced photosynthetic efficiency in presence of organic carbon sources or under mixotrophic growth (Sforza et al., 2012).

*C. sorokiniana* is considered one of the most productive species for biofuels and other bioproducts (Unkefer et al., 2017). Comparative analysis of comprehensively annotated *C. sorokiniana* complete genomes derived from three different isolates reveals significant divergence in that less than 15% of the genomes that display 80% or greater identity of nucleotide sequences among the tested strains, namely, strain 1228, UTEX 1230, and DOE 1412 (Hovde et al., 2018). Therefore, an individualized assessment of each *C. sorokiniana* strain for potential performance in cultivation systems is required (Hovde et al., 2018).

Many studies have focused on the comparison between autotrophic and mixotrophic growth of various algal species including *C. sorokiniana* (Wan et al., 2011; Li et al., 2014; Cecchin et al., 2018). The effects on cell growth and storage of energy-rich molecules upon application of different levels of supplemental organic carbon source are less clear (Li et al., 2014; Cecchin et al., 2018). Microalgae are known to increase their storage levels of energy-rich molecules such as lipid and starch in response to environmental stresses and nutrient limitation (Hu et al., 2008; Rodolfi et al., 2009). However, such enhancement is often accompanied by reduced growth leading to diminishing overall biomass productivity (Sharma et al., 2012). The two-step cultivation strategy, that is, first to generate a sufficient amount of biomass and then induce stress for accumulation of energy-rich molecules, is employed to overcome the low biomass productivity (Singh et al., 2016).

Transcriptomic profiling has been widely applied in the analysis of environmental stress responses (Gasch et al., 2000; Causton et al., 2001). Differentially expressed genes (DEGs) based on the threshold of transcription level change and *p*-value are often referred to as environmental stress factor–specific responsive genes (SRGs). This presumes that these SRGs are required for growth fitness under the stress condition tested. Growth assays using barcoded deletion strains show no correlation between SRGs and growth fitness requirements under the tested stress conditions (Giaever et al., 2002). Most of these SRGs genes turn out to be induced by the slow growth rate under suboptimal growth conditions (Regenberg et al., 2006; Brauer et al., 2008).

Although it is difficult to test the SRGs of many non-model organisms for growth fitness, change of SRG transcription levels should be correlated either positively or negatively with that of stress factor. For this reason, profiling of cells in response to reciprocal change of stress factors has allowed identification of salinity-specific responsive genes in *D. salina* (Fang et al., 2017).

Transcriptome analyses of algae in response to nutrient limitation or environmental stresses are widely applied for identification of the condition-specific response genes (Miller et al., 2010; Pillai et al., 2014; Zhu et al., 2019). While transcriptomics typically provide correlation between cellular response and phenotype at a specific time point, metabolomics reflects the real performance of the cell, defining profiles of various metabolites (Matich et al., 2019). Hence, metabolomics complements transcriptomics in the analysis of cellular responses to stress factors.

In this study, we investigated the mixotrophic growth of a novel isolate *C. sorokiniana* G32 supplemented with low (1.25 g L^–1^) and high (5 g L^–1^) levels of glucose using physiological, transcriptomic, and metabolomic analyses. We show that G32 cells accumulated energy-rich molecules, such as starch, under mixotrophic growth conditions with high glucose supplementation. We identify a subset of DEGs whose transcription level correlates with the level of glucose supplementation. These glucose-specific responsive (GSR) genes are enriched for genes involved in energy metabolism. Based on transcriptomic and metabolomic profiles, we show that excess glucose is either converted into starch or oxidized via an oxidative pentose phosphate pathway. Our analysis provides insight into mechanisms for mixotrophic growth with high glucose supplementation.

## Materials and Methods

### Algal Strains and Culture Manipulations

One of the most dominant microalgal strains in Lake Dong of Wuhan, Hubei province was isolated and cultivated in Bold’s modified Bristol (BB) or double strength 2 × BB medium (Bold, 1949) in a 1-L flask or 2-L low-form shaking flask at 100 rpm, 25°C under continuous illumination of ∼50 μmol photon m^–2^ s^–1^. The cell density of cultures was determined using either optical density (OD) at the wavelength of 680 nm or cell dry weight (CDW). For CDW analysis, approximately 50 ml of cell culture was harvested by filtration using the glass fiber filter GF/A (Whatman/GE Healthcare, Kent, United Kingdom) and dried in an oven at 80°C overnight. CDW was measured in triplicate using the AG204 balance (Mettler-Toledo Inc., Columbus, OH).

Three or four successive subcultures were done by transferring the culture at 36 h into a flask containing fresh medium to the final concentration of 0.2 OD_680_ to start the subculture. Glucose supplementation of 1.25 and 5 g L^–1^ was referred as low level and high level, respectively. Medium shift was done by spinning out the cells from the culture after at least three successive subcultures in medium supplemented with low level of glucose, washed with medium supplemented with high glucose, and resuspended in medium with high glucose at the final concentration of 0.2 OD_680_. The reciprocal medium shift was also performed. Cell samples after at least three successive subcultures with the low level and high level of glucose supplementation were designated as LL and HH, respectively. Samples 36 h after medium shift from the low level to high level and the high level to low level of glucose were designated as L2H and H2L, respectively. All samples were analyzed in triplicate.

### Sequence-Based Phylogenetic Analysis

The 18S rDNA was amplified in two overlapping fragments each with ∼900 bps in length: the upper-half fragment sequences were amplified using primers UP-FWR 5′-GCATTTGCCAAGGATGTTTT-3′ and UP-REV 5′-GGTTCACCTACGGAAACCTT-3′ and the down-half fragment using DN-FWD 5′-CTGGTTGATCCTGCCAGTAG-3′ and DN-REV 5′-CATCCTTGGCAAATGCTTTC-3′. The PCR reaction started with a pre-denaturing step at 95°C for 30 s, then entered the cycle of denaturing at 95°C for 30 s, annealing at 52°C for 30 s, and extension at 72°C for 90 s for 30 times, and finalized with extension step at 72°C for 10 min. Amplified fragments were sequenced and assembled as the G32 18S rDNA sequence (MZ266546.1).

The 18S rDNA sequences derived from various other algal species were downloaded from the NCBI database. The phylogenetic tree was constructed using MEGA (Molecular Evolutionary Genetic Analysis) software (Kumar et al., 2008).

### Determination of Starch and Lipid Contents

To determine the starch content, the Starch Assay Kit (STA-20; Sigma-Aldrich) was applied. Approximately 10 ml of the G32 liquid culture at each time point was taken for the determination of starch contents based on the procedures instructed by the manufacturer. Total lipid was extracted with methanol/chloroform (2:1 v/v) solution based on a method by Bligh and Dyer (1959) and was quantified via gravimetric analysis after evaporation of solvents. Starch and lipid contents were determined in triplicate and expressed as a percent of CDW.

### Light Microscopic and Electron Microscopic Analyses

For light microscopic analysis, a drop of fresh culture was applied to a glass slide and covered with a glass slip. Cells were examined under the upright Olympus BX53 microscope. For transmission electron microscopic analysis, cells were harvested from approximately 15 ml of culture under various growth conditions and washed with phosphate buffer or PB (in 100 ml, 530 mg of NaH_2_PO_4_⋅H_2_O, 165 mg of Na_2_HPO_4_⋅6H_2_O, pH7.0) three times. The washed cells were first fixed in 2.5% glutaraldehyde in PB for at least 4 h. After washing with PB, cells were fixed with 1% OsO_4_ in PB for 1–2 h and washed three times with PB for 15 min at each step. The fixed cells were dehydrated by a graded series of ethanol (30, 50, 70, 80, 90, and 95%) for about 15 min at each step. Subsequently, cells were dehydrated by pure alcohol for 20 min. In the end, cells were transferred to absolute acetone for 20 min. Dehydrated cells were embedded in Spurr resin (Ted Pella, Inc., Redding, CA, United States) by mixing to a graded series of acetone and Spurr resin mixture (1:1 and 1:3) for 1 h at room temperature and then to the 100% Spurr resin mixture overnight. Embedded cells were heated at 70°C for 9 h prior to ultrathin sectioning using LEICA EM UC7 ultratome (Leica Microsystems Ltd., Wetzlar, Germany). The resulting sections were stained by uranyl acetate and alkaline lead for 5 min and 10 min, respectively. Sections were analyzed using the Hitachi H-7650 instrument (Hitachi, Ltd., Japan).

### Sucrose Density Gradient Centrifugation

Step-wise sucrose gradient was prepared by overlaying 1 ml of each graded series of saturated sucrose (1.347 g ml^–1^) (Sinopharm Chemical Reagent Co., Ltd, Shanghai, China) (100, 90, 80, 70, 60, 50, 40 30, 20, and 10%) from the bottom to top of a centrifuge tube (10 mm × 104 mm, thin-wall polyallomer tube; Beckman Coulter, Inc., Atlanta, United States). One-milliliter sample (∼1.0e+08 cells) was overlaid on top of the gradient and centrifuged at 3,500 × *g* for 30 min at 4°C (Eppendorf Centrifuge 5810 R; Eppendorf AG, Hamburg, Germany). Sucrose density was determined using a refractometer (Atago R5,000; Atago USA Inc., Tokyo, Japan).

### Chlorophyll Fluorescence Analysis

Photosystem II quantum yield (Fv/Fm) of cells was determined using a chlorophyll fluorometer based on pulse amplitude modulation (PAM) technique (Maxi Imaging-PAM system; Heinz Walz GmbH, Effeltrich, Germany) by following the manufacturer’s instruction.

### RNA-Seq Data Acquisition

Total RNA from *C. sorokiniana* G32 cells was isolated using RNeasy Plant Mini Kit (Qiagen, Netherlands) according to the manufacturer’s instructions. RNA integrity number (RIN) was measured using RNA 6,000 Pico Kit (Agilent, United States) on an Agilent 2100 Bioanalyzer. High-quality RNA samples of cells prior to (LL and HH) and after (L2H and H2L) medium shift between low glucose and high glucose supplementation in triplicate were subjected to sequencing analysis using BGI PE150 platform (BGI, Beijing, China). Short-read raw sequences in FASTQ format are deposited to the NCBI Sequence Read Archive (SRA) with an accession number PRJNA744272.^1^

### *De novo* Transcriptome Assembly and Annotation

A total of ∼24 gigabases or 160 million reads (150 nt in length/read) from four time points in triplicate were pooled and subjected to *de novo* assembly using the Trinity software.^2^ FastQC (Brown et al., 2017) was used to rank the read quality and Trimmomatic (Bolger et al., 2014) was applied for removing 12 nt at the 5′-end of the read. All good reads were subjected to assembly of the transcriptome using Trinity (Grabherr et al., 2011). After removal of redundant clusters using CD-Hit (Li and Godzik, 2006) at a cutoff of similarity > 90%, a total of 32,784 non-redundant contigs were obtained. The non-redundant transcriptome were annotated based on sequence homology analysis using the Basic Local Alignment Search Tool BLASTX suite^3^ against the best proteins of the comprehensively annotated genomes of *Coccomyxa subellipsoidea*, *Chlorella variabilis*, *Chlamydomonas reinhardtii*, *Micromonas pusilla*, *Ostreococcus lucimarinus*, and *Thalassiosira pseudonana* (Armbrust et al., 2004; Merchant et al., 2007; Palenik et al., 2007; Worden et al., 2009; Blanc et al., 2010, 2012). Thus, a functional transcriptome of *C. sorokiniana* G32 that consisted of a subset of 9,722 genes (missing value is no more than one out of 12 measurements) shared homology (best-hit) to a “best” protein in the 6 algal genomes with a cutoff of *e*-value < 1.0E-07 was obtained (Supplementary Table 1).

### Analysis of Differentially Expressed Genes

To compare transcription levels between different growth conditions, read counts per gene and its normalized level of TPM (transcripts per million) were generated using RSEM software (Li and Dewey, 2011). DEGs upon increase or decrease of glucose supplementation were obtained using RSEM software (Li and Dewey, 2011) followed by EdgeR software (Robinson et al., 2010) with a cutoff of fold-change > 4-fold and FDR-corrected *p*-value < 0.01. In analysis of transcription of enzymes with multiple isoforms, summative levels were applied. Significant transcription was set at level-change > 2-fold and *p*-value < 0.05.

### Reverse-Transcriptase PCR Analysis

Reverse-transcriptase PCR analysis was applied for validation of six transcripts randomly selected from the RNA-seq analysis. Primers were designed based on the transcriptome and synthesized in BGI (BGI Genomics, Shenzhen, Guangdong, China) (Supplementary Figure 6A). Total RNA was extracted using Microalgae RNA Extraction kit (BALB Technology Co. Ltd, Beijing, China) according to the instruction by the manufacturer. cDNA was synthesized using PrimeScript Kit (Takara Bio Inc., Tokyo, Japan). The resulting cDNA was subjected to qRT-PCR (quantitative real-time PCR) using SYBR premix Ex Taq kit (Takara Bio) with the ABI 7,500 instrument (Thermo Fisher Scientific, Waltham, MA, United States). The PCR condition was as follows: initial denaturing for 2 min at 95°C, followed by 40 cycles of 15 s at 95°C and 1 min at 60°C. Correlations between RT-PCR and RNA-seq were analyzed (Supplementary Figure 6B).

### LC-MS/MS-Based Metabolomic Analysis

Metabolite extraction and LC-MS/MS-based metabolomic analysis were performed as follows: lyophilized cells (25 mg each, 6 replicates) were added with ice-cold methanol–acetonitrile–water solution (2:2:1), sonicated in ice water and ground with a steel ball, precipitated at -20°C for 2 h, and centrifuged at 25,000 rpm for 15 min at 4°C. A total of 600 μl of protein from the resulting extract was collected and dried with a refrigerated drying machine. The dried extract was dissolved and diluted with 10% methanol for mass spectrum analysis. A mixture of equal amounts of the supernatants from samples was prepared for quality control.

All chromatographic separations were performed by the LC-MS system on an ultra-performance liquid chromatography (UPLC) system (Waters Corp. Milford, MA, United States) with an ACQUITY UPLC HSS T3 column (100 mm × 2.1 mm, 1.8 μm; Waters Corp.). The column oven was maintained at 50°C. The flow rate was set as 0.4 ml min^–1^ and the mobile phase consisted of solvent A (water + 0.1% formic acid) and solvent B (acetonitrile + 0.1% formic acid). Gradient elution conditions were set as follows: 0–2 min, 100% phase A; 2–11 min, 0–100% B; 11–13 min, 100% B; 13–15 min, 0–100% A. The injection volume for each sample was 5 μl. A high-resolution tandem mass spectrometer Xevo G2 XS Q-TOF (Waters Corp.) was used to detect metabolites separated from the column. The Q-TOF was operated in both positive and negative ion modes. For positive ion mode, the capillary and sampling cone voltages were set at 3.0 kV and 40.0 V, respectively. For negative ion mode, the capillary and sampling cone voltages were set at 2.0 kV and 40.0 V, respectively. The mass spectrometry data were acquired in Centroid MSE mode. The TOF mass range was from 50 to 1,200 Da and the scan time was 0.2 s. For the MS/MS detection, all precursors were fragmented using 20–40 eV, and the scan time was 0.2 s. During the acquisition, the LE signal was acquired every 3 s to calibrate the mass accuracy.

Data processing and statistical analysis were performed as follows: The raw data were converted into CDF format using Masslynx version 4.1 (Waters Corp.) and imported into Progenesis QI software (version 2.2), which allowed the generation of a data matrix with retention time (RT), mass-to-charge ratio (m/z) values, and peak intensity. The main parameters were at default settings. The low mass ion whose relative standard deviation > 30% was removed from the data, and the QC-RLSC (Quality control-based robust LOESS signal correction) method was adopted to normalize peak intensities for comparison between samples. Putative metabolites were identified by matching the molecular mass data from redundant m/z peaks against the online HMDB,^4^ METLIN,^5^ and KEGG^6^ databases with a filter of less than 10 ppm difference between observed and theoretical mass. Then the metabolite molecular formula of matched metabolites was further evaluated by the isotopic distribution measurement (Xu et al., 2010). The resulting data were imported into the R statistic package for principal component analysis (PCA) and partial least squares-discriminant analysis (PLS-DA). Meanwhile, the corresponding variable values of importance of projection (VIP) were calculated in the PLS-DA model. The metabolomic profiles are shown in Supplementary Table 2. Threshold for significant alteration of metabolite levels was level change > 4-fold and *p*-value < 0.01.

### Statistical Analyses

Binomial test was applied to determine the non-random distribution of ESTs associated with a KEGG pathway in a subset of ESTs when compared with the presence in the whole set of ESTs. In testing transcriptional regulation of pathway flow, we first define a pathway from one compound to the other that consists of N numbers of enzymes catalyzing the reactions. The consistency of transcriptional regulation within the pathway is tested based on the number of upregulation or downregulations in a pathway. Assuming a total N number of enzymes in the pathway, M number of enzymes was upregulated or downregulated. The probability of M number of enzymes being the same upregulation or downregulation was 2^–M^. Hence, M success, N trial, and 2^–M^ probability were used for non-random binomial test using R console (R Core Team, 2018).

### Availability of Data

All raw short-read sequences in FASTQ format and *de novo* assembled non-redundant EST sequences in FASTA format supporting the conclusions of this article are available in the NCBI Sequence Read Archive (SRA) with an accession number PRJNA744272 (see text footnote 1).

## Results

### Mixotrophic Growth of a Novel Isolate Chlorella Sorokiniana G32

**FIGURE 1:**
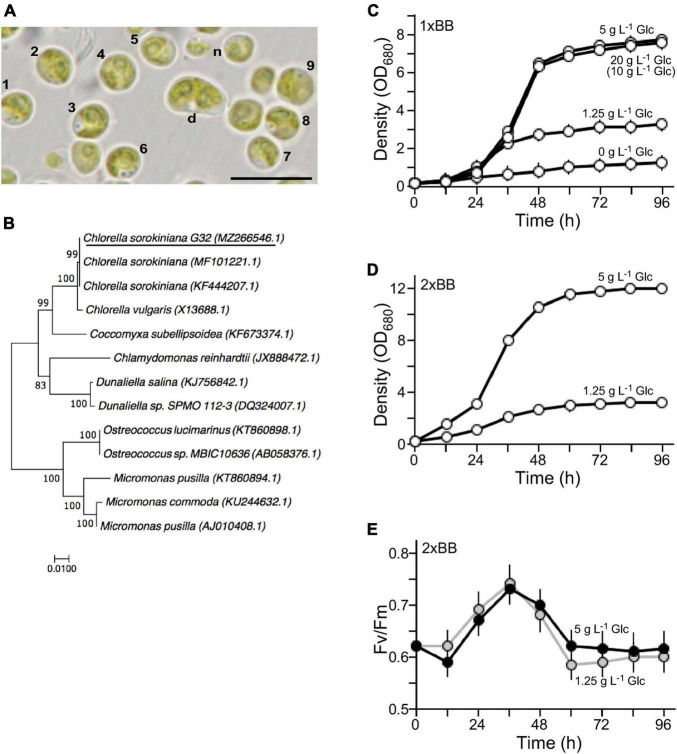
Characterization of the novel isolate G32. **(A)** Morphology of the G32 cells in 1 × BB medium supplemented with 1.25 g L^–1^ glucose. Cells with numbers are used in estimation of average cell size. n and d stand for newborn and dividing cells, respectively. A scale bar of 10 μm is shown. **(B)** Phylogenetic tree based on 18S rDNA sequence. NCBI accession numbers of sequences are shown in brackets. G32 is underlined. **(C)** Growth curves of G32 cells cultivated in 1 × BB medium supplemented with various glucose concentrations are indicated. **(D)** Growth curves of G32 cells cultivated in 2 × BB medium supplemented with 5 and 1.25 g L^–1^ glucose. **(E)** Photosynthesis efficiency of G32 cells during growth as shown in **(D)**.

The isolated G32 cells were subjected to growth in BB medium (Bold, 1949). It was greenish and round-shaped with an average diameter of ∼4.5 μm for most of the mature cells (*n* = 9) (Figure 1A). Newborn cells were relatively small and dividing cells were large (Figure 1A, see n and d). Its genomic DNA was subjected to PCR amplification for 18S rDNA sequences, analysis of which indicated that the G32 cells were most similar to that of *Chlorella sorokiniana* NON001 (MF101221.1) and KU1019 (KF444207.1) (99.9% identity) (Figure 1B). Hence, the isolate was designated as *Chlorella sorokiniana* G32, whose S18 rDNA sequence was deposited in the NCBI nucleotide sequence database with an accession number of MZ266546.1.

To investigate optimal concentrations of glucose supplement for G32 biomass production under mixotrophic condition, G32 cells were subjected to growth in BB medium supplemented with glucose at the final concentrations of 0, 1.25, 5 g, and 20 g L^–1^ under constant illumination of 50 μmol photon m^–2^ s^–1^ in a shake flask (see section “Materials and Methods”). We found that the maximum culture density without glucose supplementation was ∼1.21 OD_680_ on average (*n* = 3). On the other hand, the maximum culture densities with glucose supplementation of 1.25, 5, and 20 g L^–1^ were 2. 68-, 6. 38-, and 6.23-fold higher than that of culture without glucose supplement, respectively (*t*-test *p*-value < 0.05, *n* = 3) (Figure 1C). It appeared that cell biomass in culture supplemented with 20 g L^–1^ glucose was not any higher than that of 5 g L^–1^ glucose. As a matter of fact, it was nearly the same for 1 g L^–1^ glucose supplement. This result suggested that non-glucose nutrients in the 1 × BB medium were the limiting factor for growth.

To test this possibility, G32 cells were cultivated in double strength BB medium or 2 × BB medium. While the maximum cell density of culture supplemented with 1.25 g L^–1^ glucose was hardly altered as in 1 × BB medium, we found that culture in 2 × BB medium supplemented with 0.5% glucose reached to 12 OD_680_, which was 1.55-fold higher than that in 1 × BB medium with the same amount of glucose supplementation (*t*-test *p*-value < 0.05, *n* = 3) (Figure 1D).

To investigate whether addition of glucose in culture would affect photosynthesis, we assessed the photosystem II efficiency (Fv/Fm) based on chlorophyll fluorescence in cultures during growth (see section “Materials and Methods”). It was clear that efficiency of PSII in 2 × BB culture without glucose supplementation (i.e., phototrophic growth) was identical to that of culture supplemented with 1.25 g L^–1^ glucose (i.e., mixotrophic growth), suggesting that photosynthesis was unaffected upon addition of glucose under mixotrophic growth (Supplementary Figure 2). We found that when level of glucose supplementation was increased to 5 g L^–1^, photosynthesis efficiency was slightly reduced prior to the peak of photosynthesis efficiency at 36 h compared with that of the culture supplemented with 1.25 g L^–1^ glucose (Figure 1E). The photosynthesis efficiency of culture with 5 g L^–1^ glucose supplementation was a bit increased after its peak level compared with that of the culture with 1.25 g L^–1^ glucose. This result indicated that the photosynthesis efficiency in G32 mixotrophic growth was uncompromised as it is in phototrophic growth.

### High Cell-Mass Density in G32 Cultures With High Glucose Supplementation

**FIGURE 2:**
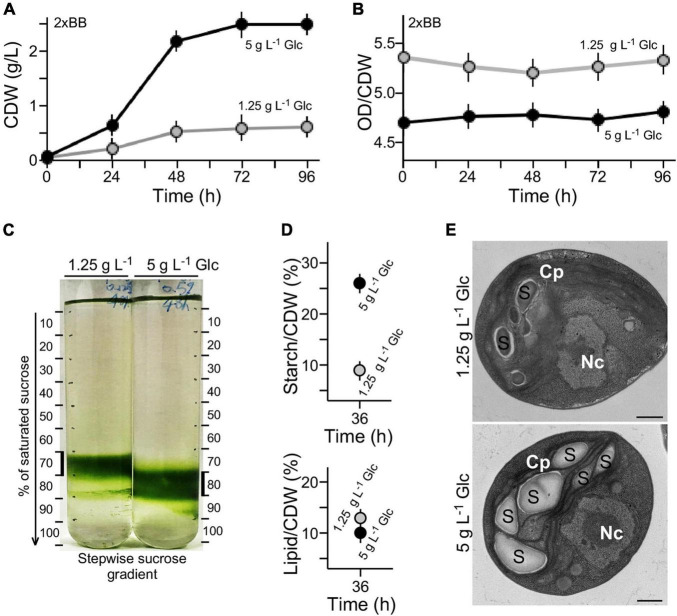
High cell density is associated with high-level accumulation of starch in medium with high glucose supplementation. **(A)** Growth curves expressed in CDW. **(B)** Conversion rate between OD value and CDW of cultures with high glucose and low glucose supplementation. **(C)** Sucrose gradient centrifugation analysis of cell density in medium with high glucose and low glucose supplementation. **(D)** Starch and lipid contents of G32 cells with high glucose and low glucose supplementation. **(E)** Transmission electron microscopic analysis. Nc and Cp stand for nucleus and chloroplast, S for starch granule. Scale bar of 500 nm is shown.

Growth curves expressed in OD_680_ units (see Figure 1D) were also analyzed using cell dry weight (CDW) (see section “Materials and Methods”) (Figure 2A). We found that the conversion rate of OD_680_ value over CDW g L^–1^ value in 2 × BB culture supplemented with 1.25 g L^–1^ glucose was approximately 5.28 on average (Figure 2B). On the other hand, the conversion rate in 2 × BB culture supplemented with 5 g L^–1^ glucose was 4.75 on average, 10% lower than that with low glucose (*t*-test *p*-value < 0.05, *n* = 3). The result suggests that more cell biomass in culture with high glucose supplementation (i.e., 5 g L^–1^ glucose) was produced than with low glucose (i.e., 1.25 g L^–1^ glucose). To test this possibility, we investigated the cell-mass density using stepwise sucrose density gradient centrifugation. The analysis showed that cells in cultures with high glucose supplementation exhibited higher density than those with low glucose (Figure 2C).

Starch granules are high-density constituents in cells, whereas lipids are low-density molecules (Hathwaik et al., 2015). To investigate whether the different cell-mass densities were associated with different levels of starch or lipid content in cell mass, levels of starch and total lipid on a CDW basis were determined (see section “Materials and Methods”). We found that the starch level in culture with high glucose supplementation was approximately 2.8-fold higher than that with low glucose (*t*-test *p*-value < 0.05, *n* = 3) (Figure 2D). On the other hand, we found that lipid content in cells with low glucose supplementation was only mildly increased by 1.2-fold compared with that with high glucose (*t*-test *p*-value > 0.05, *n* = 3). These results suggested that high starch content in cells with high glucose supplementation was the major cause for increased cell-mass density compared with that with low glucose. This was further supported by the EM analysis showing more starch granules accumulated in cells with high glucose supplementation than that with low glucose (Figure 2E).

### Glucose-Specific Responsive Genes Revealed by Transcriptomic Analysis in G32 Cells

**FIGURE 3:**
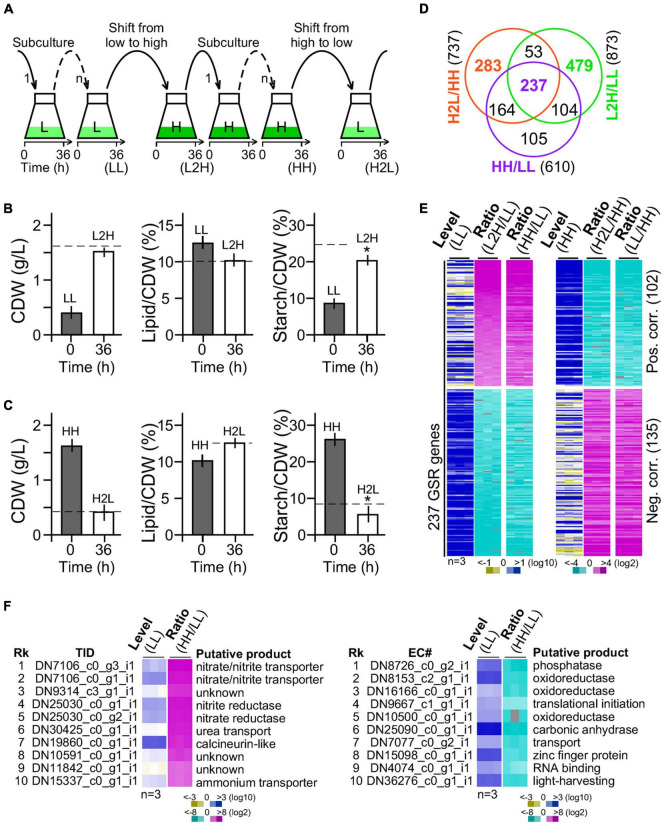
Transcriptomic profiling of G32 cells maintained in and shifted to medium with different levels of glucose supplementation. **(A)** Schematic drawing of G32 culture maintenance and medium shift. Subculture in low-glucose and high-glucose media and shift to medium with different levels of glucose supplementation at 36 h after inoculation. **(B)** Bar plots showing the CDW, lipid, and starch contents upon medium shift from low glucose to high glucose supplementation (L2H). Dashed line indicates the level in cells adapted to high glucose supplementation (HH). Asterisk “*” indicates the statistical significance of difference between starch levels in L2H and HH samples. **(C)** Bar plots showing the CDW, starch, and lipid contents upon medium shift from high to low glucose supplement (H2L). Dashed line indicates the level in cells adapted to low glucose supplement levels (LL). **(D)** Venn diagram of DEGs between L2H/LL, H2L/HH, and HH/LL. **(E)** Heatmap of the 237 GSR genes profile. Level and ratio are shown. Color keys are shown at the bottom. **(F)** Top 10 most upregulated (left panel) and downregulated (right panel) genes. Rk and TID stand for rank and transcript ID, respectively.

We wanted to investigate the glucose-specific response genes in cells upon change of glucose supplement levels. GSR genes were expected to display significantly altered transcription levels that are positively or negatively correlated with the amount of glucose supplementation. Hence, G32 cells after adaptation in medium with high and low glucose supplementation (i.e., at least six generations by three or more successive subculturing) were subjected to medium shift from low to high glucose supplementation or high to low glucose supplementation (Figure 3A).

We found that CDW and lipid content in cultures 36 h after medium shift from low glucose to high glucose supplementation were nearly identical to that adapted to high glucose supplementation (Figure 3B, left and middle panels). However, starch content in culture 36 h after medium shift from low glucose to high glucose supplementation was 20% less than that adapted to high glucose supplementation (*t*-test *p*-value < 0.05, *n* = 3) (Figure 3B, right panel). Similarly, CDW and lipid content in cells 36 h after medium shift from high glucose to low glucose supplementation were identical to that adapted to low glucose supplementation (Figure 3C, left and middle panels). Notably, we found that the starch level in cells 36 h after medium shift from high glucose to low glucose supplementation was 30% lower than that adapted to low glucose supplementation (*t*-test *p*-value < 0.05, *n* = 3) (Figure 3C, right panel), suggesting an overreaction in starch hydrolysis upon medium shift. This result indicated that adjustment of starch content was a bit slow upon change of glucose supplement compared with lipid content and CDW.

Cells adapted to low glucose (LL) and high glucose (HH) supplementation and 36 h after medium shift from low glucose to high glucose supplementation (L2H) and from high glucose to low glucose (H2L) supplementation were subjected to RNA-seq analysis using PE150 platform in triplicate (BGI Genomics, Shenzhen, Guangdong, China) (see section “Materials and Methods”). A total of 24 gigabases or 160 million reads from 12 samples (i.e., a total of four conditions, each with three repeats) were obtained (available in SRA database with an accession number of PRJNA744272). A *de novo* transcriptome using the Trinity software (Grabherr et al., 2011) was assembled with a set of annotated 9,277 genes (Supplementary Table 1 and Supplementary Figure 3, see section “Materials and Methods”).

Out of a total of 9,277 genes, 873 (9.41%) and 737 (7.94%) were DEGs (level change > 4-fold, FDR corrected *p*-value < 0.01) in G32 cells 36 h after medium shift from low glucose to high glucose supplementation and from high glucose to low glucose supplementation, respectively (Figure 3D). We found that 290 genes were common in significant response to both changes of glucose supplement levels, majority (237 or 81.7% genes) of which exhibited monotonic alteration or remained to be significantly altered in low glucose and high glucose supplementation (Figure 3E). Transcriptional response of the 237 common DEGs was either positively or negatively correlated with the level of glucose supplementation, implying that they were GSR genes. In the top 10 most upregulated GSR genes, nitrite/nitrate/ammonium transporter and nitrite/nitrate reductase involved in nitrogen metabolisms (p00910) were enriched by 250-fold compared with that of background level (binomial test *p*-value < 2.2e-16) (Figure 3F, left panel). On the other hand, in the top 10 most downregulated GSR genes, oxidoreductases were enriched by 35-fold compared with that of background (binomial test *p*-value < 2.2e-16) (Figure 3F, right panel). Functional enrichment analysis indicated that genes associated with nitrogen metabolism, thiamine metabolism, arginine biosynthesis, carbon fixation, and ribosome biogenesis were overrepresented in the subset of the GSR genes (binomial test *p*-value < 0.05) (Table 1). These results indicated that GSR genes are enriched for energy-related metabolisms.

**TABLE 1:** Functions enriched in the subset of glucose-specific responsive (GSR) genes.

PID^a^	Pathway	Genome^b^	GSR^c^	FC^d^	*p*-value*^e^*
p00910	Nitrogen metabolism	18	8	4.12	3.00E-08
p00730	Thiamine metabolism	22	4	2.83	2.61E-03
p00220	Arginine biosynthesis	23	4	2.77	3.06E-03
p00710	Carbon fixation	51	8	2.62	6.00E-05
p03008	Ribosome biogenesis	83	10	2.24	6.65E-05
All	9,277	237			

*^a^Pathway ID.*

*^b^Number of the pathway-associated genes in the genome or transcriptome.*

*^c^Number of the pathway-associated genes in the subset of the GSR genes.*

*^d^Fold-change in log2 scale; e, binomial test p-value for non-random distribution.*

### Many Compounds Identified Are Involved in Various KEGG Metabolic Pathways

**FIGURE 4:**
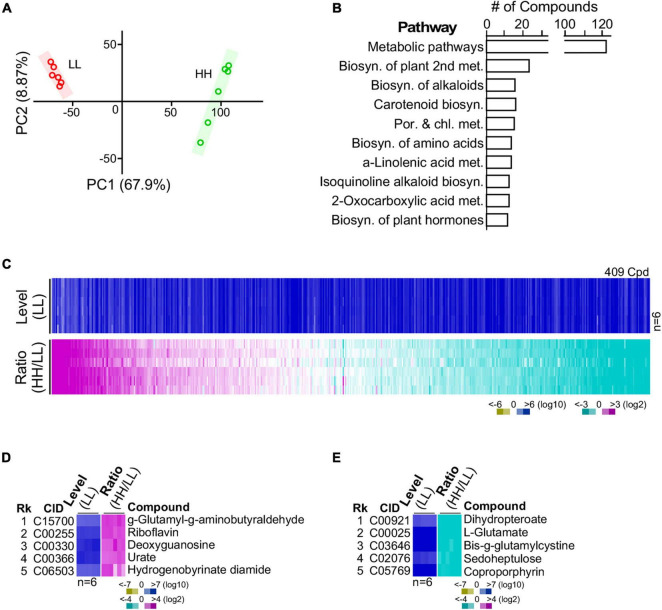
Metabolite profiling of G32 cells maintained in media supplemented with high-level and low-level glucose. **(A)** High correlation between profiles of repeated experiments using principal component analysis. **(B)** Top 10 numbers of pathway-associated metabolites. **(C)** Profile of metabolites in cells cultivated with high and low glucose supplements. **(D)** Top five upregulated metabolites. **(E)** Top five downregulated metabolites.

G32 cells adapted with high glucose and low glucose supplementation were subjected to LC-MS/MS-based metabolomic analysis in sextuplicate with good correlation (Figure 4A) (see section “Materials and Methods”). After selection of high-quality single-molecule ions (score > 35; fraction score > 20), a set of 409 metabolites was obtained, 30% of which possessed annotation in KEGG metabolic pathways (Figures 4B,C and Supplementary Table 2). Approximately 18% of the 409 metabolites was differentially expressed compounds or DECs (i.e., level change > 4-fold and *p*-value < 0.01). Among the top five most upregulated metabolites, the most upregulated molecule γ-glutamyl-γ-aminobutyraldehyde was involved in the arginine and proline metabolism (Figure 4D). The second, riboflavin, was involved in riboflavin metabolism. The third (deoxyguanosine) and fourth (urate) were involved in purine metabolism. Also, the fifth most upregulated molecule, hydrogenobyrinate diamide, was involved in porphyrin and chlorophyll metabolism. On the other hand, the top most downregulated molecules dihydropteroate, L-glutamate, oxidized gamma-glutamylcysteine, sedoheptulose, and coproporphyrin were involved in folate biosynthesis, arginine biosynthesis, glutathione metabolism, carbon fixation, and porphyrin and chlorophyll metabolism, respectively (Figure 4E). Some of these functions such as arginine biosynthesis and carbon fixation associated with the most altered metabolites were also found to be enriched by the GSR genes (see Table 1).

### Glucose Is Assimilated Through the Oxidative Pentose Phosphate Pathway

**FIGURE 5:**
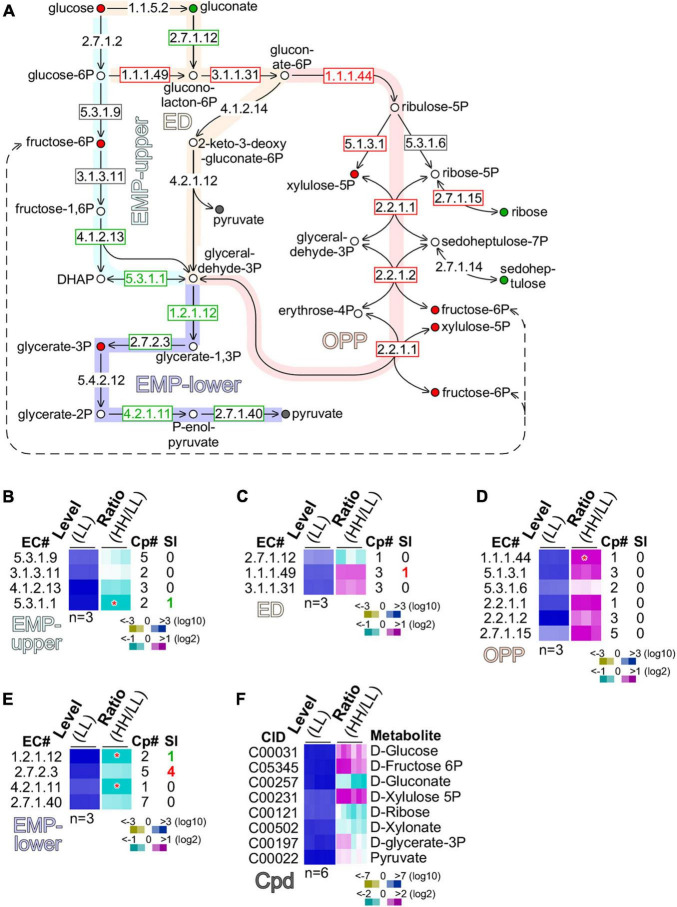
Glucose is preferentially oxidized through the oxidative pentose phosphate (OPP) pathway. **(A)** Schematic drawing of the EMP (Embden–Meyerhorf–Parnas)-upper pathway, ED (Entner–Doudoroff) pathway, oxidative pentose phosphate (OPP) pathway, and EMP-lower pathway. Enzyme EC numbers with rectangular outline are shown. Enzymes without rectangular outline are not found in transcriptome. Significant (level change > 4-fold, *p*-value < 0.01) and moderate (level change > 1.2-fold) changes of enzyme levels are color coded with red, gray, and green of EC number and rectangular outline for upregulation, no change, and downregulation, respectively. Metabolites are shown as circle with color fill. Metabolites without color fill or white fill are not found in metabolome. Significant (level change > 4-fold, *p*-value < 0.01) and moderate (level change > 1.2-fold) changes of metabolite levels are indicated with red, gray, and green of circle outline and inner fill for upregulation, no change, and downregulation, respectively. **(B)** Heatmap of transcription level change of enzymes involved in MEP-upper. Heatmap is based on summative level of enzyme isoforms when available. Asterisk “*” sign indicates a significant change of summative levels (level change > 2-fold, *p*-value < 0.05) change. Magenta, white, and cyan stand for upregulation, unchanged, and downregulation of transcription, respectively. Total number of isoform copies (Cp#) is shown. Number of significantly changed isoforms (SI) is also shown. Color keys are shown in the bottom. Heatmap of profiles of enzymes involved in **(C)** ED, **(D)** OPP, and **(E)** EMP-lower are shown. The display is identical to **(B)**. **(F)** Heatmap of profiles of metabolites involved in the above pathways is shown. CID stands for compound ID.

To investigate metabolic pathways utilized for glucose assimilation in culture with high glucose supplementation, we examined transcripts and metabolites involved in glycolysis [the Embden–Meyerhorf–Parnas (EMP) pathway], the Entner–Doudoroff (ED) pathway, and the oxidative pentose phosphate pathway (Figure 5A). There were four enzymes, namely, the glucose-6-phosphate isomerase (GPI, EC 5.3.1.9), fructose-bisphosphatase (FBP, EC 3.1.3.11), fructose-bisphosphate aldolase (FBPAL, EC 4.1.2.13), and triose-phosphate isomerase (TPI, EC 5.3.1.1) found in the upper part of the EMP (or EMP-upper) pathway from glucose to glyceraldehyde-3-phosphate. While transcription level of GPI (EC 5.3.1.9) and FBP (EC 3.1.3.11) was unchanged, FBPAL (EC4.1.2.13) and TPI (EC 5.3.1.1) were downregulated moderately (level change > 1.2-fold) and significantly (level change > 2-fold, *p*-value < 0.05), respectively. This result suggests that the EMP-upper pathway in cells with high glucose supplementation was not upregulated compared with that with low glucose (Figure 5B).

Although a number of enzymes (EC 1.1.5.2; EC 4.1.2.14; EC 4.2.1.12) in the ED pathway were missing in the transcriptome, we found that gluconokinase (GCK, EC 2.7.1.12) was moderately downregulated (level change > 1.2-fold), while glucose-6-phosphate dehydrogenase (GPDH, EC 1.1.1.49) and 6-phosphogluconolactonase (PGCL, EC 3.1.1.31) were moderately upregulated (level change > 1.2-fold) (Figure 5C). This result suggests that glucose was preferentially converted into gluconolacton-6-phosphate via glucose-6-phosphate, but not gluconate.

In the OPP pathway, five out of six enzymes, namely, the phosphogluconate dehydrogenase (PGCDH, EC 1.1.1.44), ribulose-phosphate 3-epimerase (RBPE, EC 5.1.3.1), ribose-5-phosphate isomerase (RBPI, EC 5.3.1.6), transketolase (TKT, EC 2.2.1.1), transaldolase (TAL, EC 2.2.1.2), and ribokinase (RBK, 2.7.1.15), exhibited significant or moderate upregulation (*p*-value = 1.74E-07), suggesting that the OPP pathway was the primary route for glucose oxidation (Figure 5D). In addition, we found that based on the summative levels, four enzymes such as glyceraldehyde-3-phosphate dehydrogenase (GAPDH, EC 1.2.1.12), phosphoglycerate kinase (PGK, EC 2.7.2.3), phosphopyruvate hydratase (PPH, EC 4.2.1.11), and pyruvate kinase (PK, EC 2.7.1.40) in the low part of the EMP (or EMP-lower) pathway were either significantly or moderately downregulated (Figure 5E). Although the summative level of PGK (EC 2.7.2.3) was moderately downregulated, we found that four out of the five PGK isoforms were actually significantly upregulated (see Figure 5E). This discrepancy could be caused by the non-identical functions of the five isoforms of PGK.

To circumvent this issue, we investigated the level change of various metabolites involved in glucose assimilation (Figure 5F). Consistent with the level of glucose supplementation, glucose content in cells with high glucose supplementation was moderately increased compared with that with low glucose. Downregulation of gluconate was consistent with the notion that glucose assimilation underwent via glucose-6-phasphate rather than gluconate. Upregulation of xylulose-5-phosphate and fructose-6-phosphate was in agreement with the upregulation of enzymes in the OPP pathway. Significantly, we found that glycerate-3-phosphate was upregulated, consistent with the upregulation of the four PGK isoforms. This result suggests that the EMP-lower pathway was activated for glucose assimilation in cells with high glucose supplementation.

### Starch Biosynthesis and Energy Metabolisms Are Upregulated in Cells With High Glucose Supplementation

**FIGURE 6:**
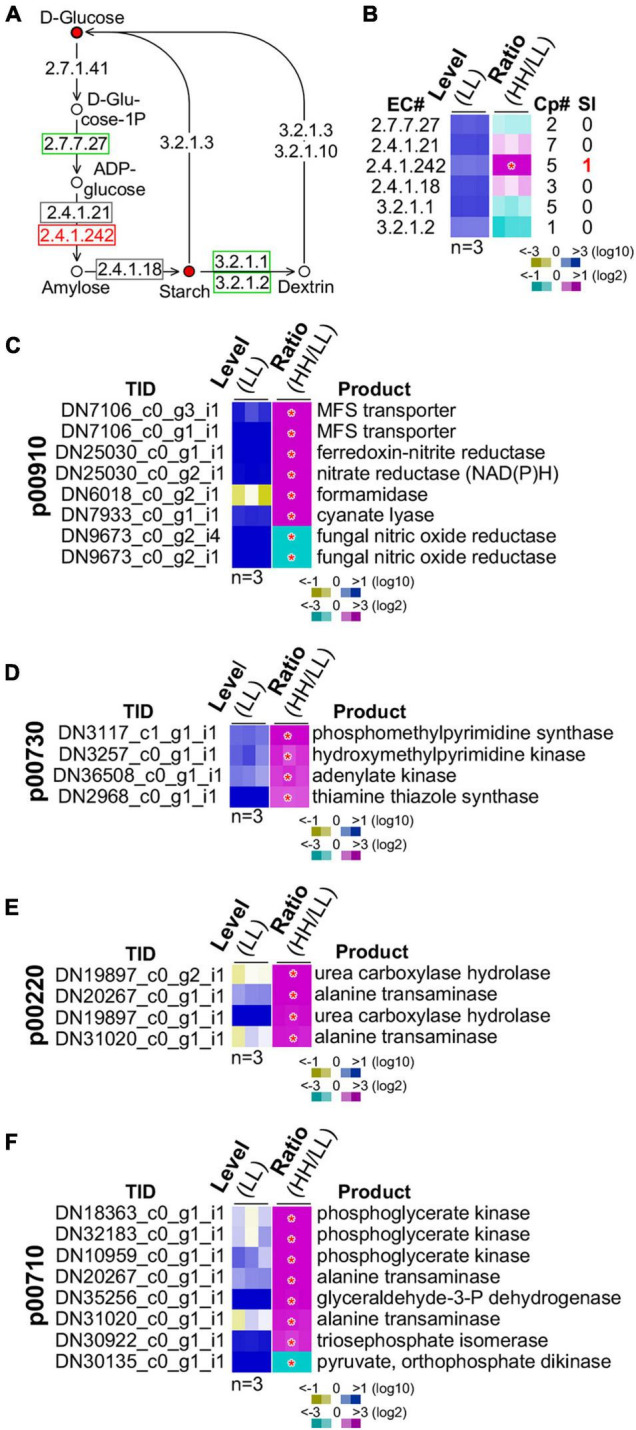
Regulation of starch biosynthesis and GSR genes enriched functions. **(A)** Schematic drawing of the starch biosynthesis pathway. The display is identical to Figure 5A. **(B)** Heatmap of transcription level change of enzymes involved in starch biosynthesis. The display is identical to Figure 5B. Heatmaps of profiles of GSR genes involved in **(C)** nitrogen metabolism (p00910), **(D)** thiamine metabolism (p00730), **(E)** arginine metabolism (p00220), and **(F)** carbon fixation (p00710). Transcript ID and its putative product are shown. The display is identical to **(B)**.

We subsequently investigated the starch biosynthesis pathway (Figure 6A). It was clear that intracellular glucose content in culture with high glucose supplementation was higher than that with low glucose (see Figure 5F). We found that the summative level of the five isoforms of the granule-bound starch synthase (GBSS, EC 2.4.1.242) was significantly upregulated (level change > 2-fold, *p*-value < 0.05) (Figure 6B). One of the five GBSS isoforms was also significantly upregulated (level change > 4-fold, *p*-value < 0.01). These results suggested that BGSS could play a key role in regulation of starch accumulation with high glucose supplementation.

A number of energy metabolisms were enriched in the GSR genes (see Table 1). In the nitrogen metabolism, six out of eight GSR genes exhibited positive correlation with the level of glucose supplementation (*p*-value = 3.9E-10) (Figure 6C). In the thiamine metabolism and arginine biosynthesis, all four GSR genes were upregulated (*p*-value = 1.5E-05) (Figures 6D,E). In addition, of the eight GSR genes in the carbon fixation pathway, seven were positively correlated with the level of glucose supplementation (*p*-value = 1.5E-14) (Figure 6F). These results implied that high glucose supplementation promoted energy metabolisms resulting in rapid growth rate and accumulation of energy-rich starch.

However, we noticed that in the ribosome biogenesis pathway, all of the 10 GSR genes exhibited negative correlation with the level of glucose supplementation (*p*-value = 2.2E-16) (Supplementary Figure 4). This might represent the fraction of transcripts that was inactivated through heterochromatin formation as found in mammalian systems (Santoro et al., 2002; Murayama et al., 2008). In mammal, ribosome biogenesis is primarily controlled at the post-transcriptional level (Lempiainen and Shore, 2009).

## Discussion

It is known that accumulation of energy-rich molecules in microalgae often occurs at the cost of overall biomass productivity (Sharma et al., 2012). To overcome the low productivity, a two-step strategy in which it first increases biomass and then induces accumulation of energy-rich molecules by stress has been proposed for production of energy-rich molecules such as starch and lipid (Singh et al., 2016). In this study, we show that, in the shake-flask setting, both growth rate and accumulation of starch increase in *C. sorokiniana* G32 under mixotrophic growth condition with 5 g L^–1^ glucose supplementation compared with that of 1.25 g L^–1^ glucose. This result indicates that increasing accumulation of energy-rich molecules in algae cells can be accompanied by increased biomass productivity in *C. sorokiniana* G32 under mixotrophic growth.

Mixotrophy is the ability to combine autotrophic and heterotrophic modes of nutrition. Mixotrophic microorganisms are widely spread in ecosystems, which complicates the concept of the materials and energy flow in aquatic ecosystems (Matantseva and Skarlato, 2013). In this study, we show that without presence of organic carbon source, *C. sorokiniana* G32 grows autotrophically. Upon availability of organic carbon such as glucose, it undergoes both autotrophically and heterotrophically (see Figure 1). In mixotrophic growth, we find that heterotrophic growth can predominate when organic carbon source suffices. This character makes it attractive for rapid biomass production (Wan et al., 2011; Li et al., 2014; Cecchin et al., 2018). In this study, by using a shake-flask growth mode, we show that *C. sorokiniana* G32 reaches the maximum growth rate of 1.22 day^–1^ and biomass productivity of 1.55 g L^–1^ day^–1^ in medium supplemented with 5 g L^–1^ glucose (see Figure 2A and Supplementary Figure 5A), in which starch and lipid contents are 26 and 10% of CDW, respectively (see Figure 2D). In a study by Wan et al. (2011), the authors show that *C. sorokiniana* M209220 exhibits the maximum growth rate of 0.49 day^–1^ and productivity of 0.33 g L^–1^ day^–1^ with supplementation of 10 g L^–1^ glucose. Although the growth rate and productivity appear to be relatively low, the lipid content is as high as 50% of CDW. On the other hand, Li et al. (2014) show that *C. sorokiniana* UTEX1602 displays a relatively high growth rate (i.e., 1.32 day^–1^) and productivity (i.e., 2.2 g L^–1^ day^–1^) with 4 g L^–1^ glucose supplementation. Under this condition, lipid content reaches 32%. When glucose supplementation increases to 6 g L^–1^, although lipid content is hardly altered, growth rate and productivity are increased to 1.69 day^–1^ and 3.4 g L^–1^ day^–1^, respectively. Li et al. (2014) show that optimal light intensity is 100 μmol m^–2^ s^–1^ in mixotrophic growth. Besides, CO_2_ supply can significantly increase the productivity. We find that productivity of G32 is hardly increased by doubling the light intensity to 100 μmol m^–2^ s^–1^ (Supplementary Figure 5B). However, by not only increasing the light intensity to 100 μmol m^–2^ s^–1^, but also the glucose supply to 6 g L^–1^ and BB medium strength to 3-fold, the productivity of G32 reaches 3.55 g L^–1^ day^–1^, higher than the productivity of 3.4 g L^–1^ day^–1^, one of the best previously reported using the similar shake-flask growth system (Li et al., 2014). Hence, we propose that *C. sorokiniana* G32 is suitable for algal biomass production.

Mixotrophic growth can significantly promote algal biomass productivity (Wan et al., 2011; Li et al., 2014; Cecchin et al., 2018). However, the significant cost of glucose in the total cost of mixotrophic algal biomass production is a major drawback. Low-cost carbon source from industrial and agricultural waster such as corn powder hydrolysate and sugar cane molasses has been employed to replace glucose in algal mixotrophic growth (Xu et al., 2006; Sousa et al., 2014). While algae-based lipid is suitable for biodiesel production, algae-based starch is a useful feedstock for bioethanol production (Chisti, 2007; Hu et al., 2008; Ho et al., 2013).

In a photobioreactor (PBR) growth system, Cecchin et al. (2018) have shown that a number of enzymes involved in carbon fixation such as glyceraldehyde 3-phosphate dehydrogenase (GAPDH, EC 1.2.1.12), pyruvate orthophosphate dikinase (PPDK, EC 2.7.9.1), and aspartate aminotransferase (ASPAT, EC 2.6.1.1) whose activity under mixotrophic condition is downregulated compared with that of phototropic condition, while phosphoenolpyruvate carboxylase (PPC, EC 4.1.1.31) is upregulated (Cecchin et al., 2018). We have compared the transcriptional level of these enzymes under high glucose and low glucose supplementation. Based on the summative level of the isoforms, downregulation of GAPDH, PPDK, and ASPAT and upregulation of PPC also occur between high glucose and low glucose supplementation (Supplementary Figure 6). This result implies that transcriptional re-programming in *C. sorokiniana* between phototrophic growth and acetate-based mixotrophic growth is similar to that between mixotrophic growth conditions with low glucose and high glucose supplementation.

Granule-bound starch synthase (GBSS, EC 2.4.1.242) in rice is responsible for amylose synthesis in rice seed endosperm (Liu et al., 2014). By using Cas9–gRNA RNP complex, Andersson et al. (2018) have knocked out the potato GBSS yielding an exogenous DNA-free amylose-free potato. In our analysis, we show that GBSS plays a pivotal role in accumulation of starch granules in *C. sorokiniana* G32 under high glucose supplementation, consistent with the observation made in rice (Liu et al., 2014).

In this study, we show that GSR genes are enriched for ribosome biogenesis, nitrogen metabolism, thiamine metabolism, and carbon fixation (see Table 1). While majority of the GSR genes involved in nitrogen metabolism, carbon fixation, and thiamine metabolism display a positive correlation with the level of glucose supplementation (see Figure 6), all of the 10 GSR genes involved in ribosome biogenesis show inversed correlation with that of glucose supplementation (see Supplementary Figure 4). We show that cells with high glucose supplementation exhibit higher growth rate and maximum cell density than with low glucose, indicating the high level of energy status in cell with high glucose supplementation (see Figure 1). Consistent with this, genes involved in energy metabolisms such as nitrogen metabolism and carbon fixation are significantly upregulated, correlating with the level of glucose supplementation or energy status. Conversely, transcriptional activities of genes involved in ribosome biogenesis are negatively correlated with the level of glucose supplementation. Ribosome biogenesis is important for cell growth, which adapts to changes in intracellular energy status (Lempiainen and Shore, 2009). Nevertheless, in rapid growing mammalian cells, not all rDNA copies are in an active state. Santoro et al. (2002) have shown that about half of the rDNA copies are silenced through an epigenetic layer of RNAP I regulation that is associated with a chromatin remodeling complex NoRC (Nucleolar Remodeling Complex). A fraction of active rDNA copies are proposed to be linked to an rDNA corepressor eNoSC (energy-dependent Nucleolar Silencing Complex) (Murayama et al., 2008). A similar mechanism for regulation of genes involved in ribosome biogenesis may occur in *C. sorokiniana*.

## Conclusion

In conclusion, we show that *C. sorokiniana* G32 accumulates energy-rich molecule starch without impeding growth rate and biomass productivity when supplemented with high-level glucose. By combining the transcriptome and metabolome profiling, we show that assimilation of glucose in cell with high glucose supplementation is preferentially undertaken via oxidative pentose phosphate pathway. Furthermore, we identify a subset of GSR genes whose transcriptional alteration is correlated with the level of glucose supplementation. GSR genes are enriched for energy metabolic pathways and likely to play a major role in regulation of cell growth rate and biomass productivity. Moreover, we show that granule-bound starch synthase (GBSS) may play a key role in regulation of starch accumulation under the mixotrophic growth condition. We propose that *C. sorokiniana* G32 has potential for biomass production.

## Data Availability Statement

The datasets presented in this study can be found in online repositories. The names of the repository/repositories and accession number(s) can be found below: https://www.ncbi.nlm.nih.gov/, PRJNA744272.

## Author Contributions

QZ, MZ, BL, and ZY carried out the biological and biochemical studies. FW, QZ, ZY, and JL carried out the bioinformatics and statistical studies. JL conceived of the study and participated in its design and coordination, and drafted the manuscript. All authors read and approved the final manuscript.

## Conflict of Interest

The authors declare that the research was conducted in the absence of any commercial or financial relationships that could be construed as a potential conflict of interest.

## Publisher’s Note

All claims expressed in this article are solely those of the authors and do not necessarily represent those of their affiliated organizations, or those of the publisher, the editors and the reviewers. Any product that may be evaluated in this article, or claim that may be made by its manufacturer, is not guaranteed or endorsed by the publisher.

## Footnotes

1
http://ncbi.nlm.nih.gov/sra

2
http://trinityrnaseq.github.io

3
http://blast.ncbi.nlm.nih.gov

4
http://www.hmdb.ca/

5
http://metlin.scripps.edu/

6
http://www.genome.jp/kegg/
