# Supplementary material for: Transcriptome and Metabolome Profiling of a Novel Isolate Chlorella sorokiniana G32 (Chlorophyta) Displaying Enhanced Starch Accumulation at High Growth Rate Under Mixotrophic Condition
Source: Front Microbiol. 2022 Jan 6;12:760307. doi: 10.3389/fmicb.2021.760307 (PMC8770532; doi:10.3389/fmicb.2021.760307)

Supplementary Figure S1. Correlation of transcription levels between RNA-seq and qRT-PCR

(A) Primer sequences for qRT-PCR

**TID**	**FWD Primer**	**REV Primer**
DN14845_c0_g1_i1	TATCGACCGCATGTGGGA	CTTGGTGGCAAAGAACTGG
DN14904_c0_g1_i1	GTGGTGGTCAAGTCCTTTGC	GATCTTGTCAAAGGGACCCA
DN25030_c0_g1_i1	CTTGATGGAGAAGTAGCCGC	TTCATGAGTGGGATGGACAA
DN2914_c0_g1_i1	AGATCGACTCCCTGTATGAGG	TGCCGTTGAAGAAGTCCTG
DN30203_c0_g1_i1	CAACGAGATGGCCGAGGTG	CCATCATGGAGAAGTTGTAGCC
DN3105_c0_g1_i1	TCATCTGGTTTGTGGACGAC	CGGGTTGAAGATGAAGATGG

(B) Dot plot showing correlation between RNA-seq and qRT-PCR analyses. Ratios in log 2 scale of six genes in (A) are shown.

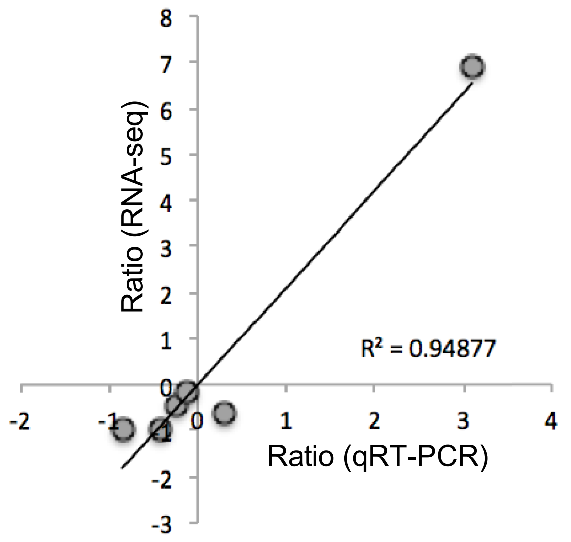

Supplement: Supplementary file 1 [file Data_Sheet_1.PDF]
