# Supplementary material for: Transcriptome and Metabolome Profiling of a Novel Isolate Chlorella sorokiniana G32 (Chlorophyta) Displaying Enhanced Starch Accumulation at High Growth Rate Under Mixotrophic Condition
Source: Front Microbiol. 2022 Jan 6;12:760307. doi: 10.3389/fmicb.2021.760307 (PMC8770532; doi:10.3389/fmicb.2021.760307)

Supplementary Figure S3. Characteristics of the G32 transcriptome. (A) Number of best-hit in the annotated transcriptome. (B) Correlation between RNA-seq repeats; (C) Histogram of gene sizes. (D) Histogram of gene transcription levels. (E) Top ten metabolic pathways in the transcriptome. (F) Top ten pathways involved in genetic information processing.

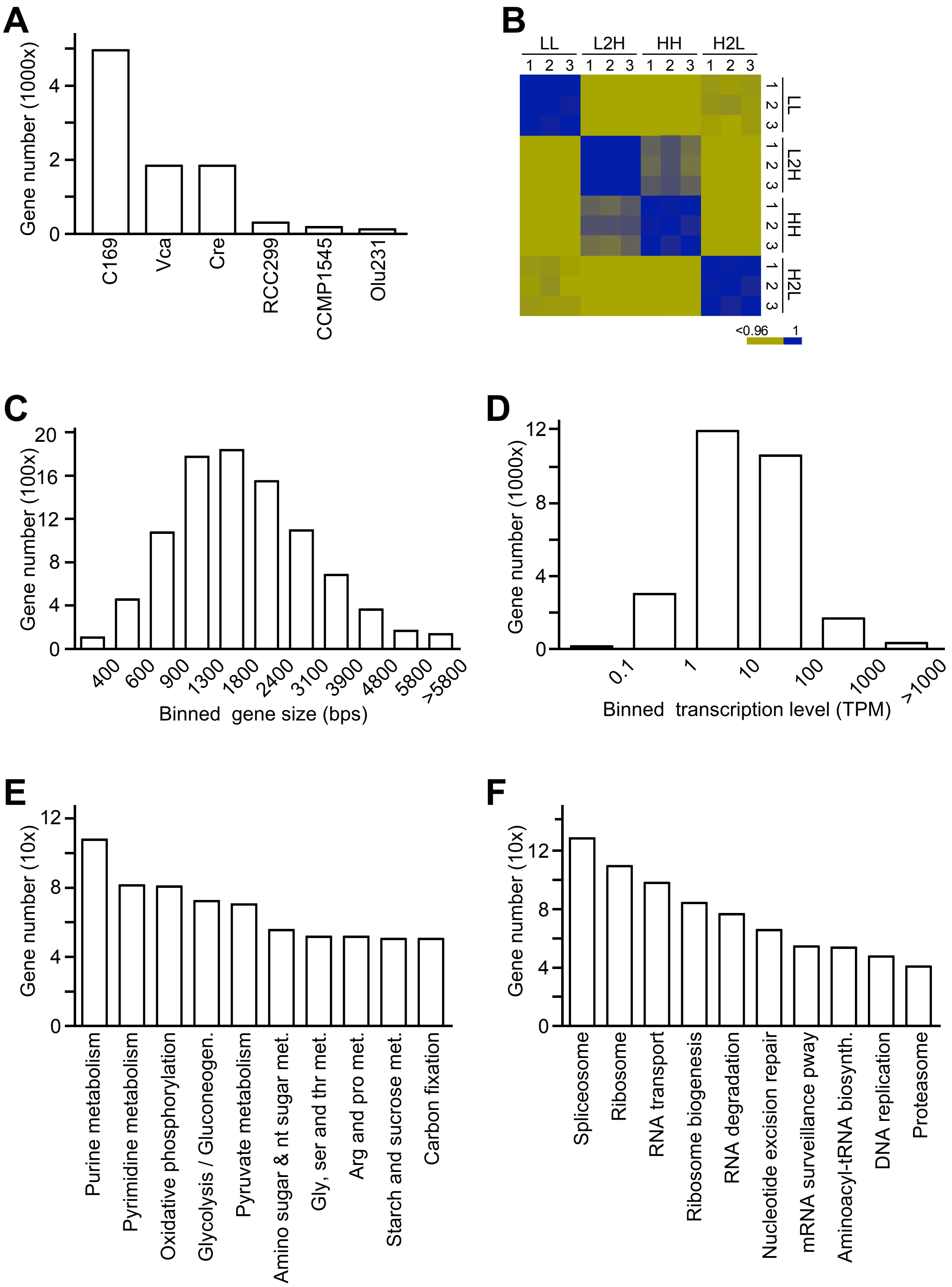

Supplement: Supplementary file 3 [file Data_Sheet_3.PDF]
