# Supplementary figures and images for: Transcriptome and Metabolome Profiling of a Novel Isolate Chlorella sorokiniana G32 (Chlorophyta) Displaying Enhanced Starch Accumulation at High Growth Rate Under Mixotrophic Condition
Source: Front Microbiol. 2022 Jan 6;12:760307. doi: 10.3389/fmicb.2021.760307 (PMC8770532; doi:10.3389/fmicb.2021.760307)

**Supplementary Figure S4.** Heatmap of GSR genes associated with ribosome biogenesis.

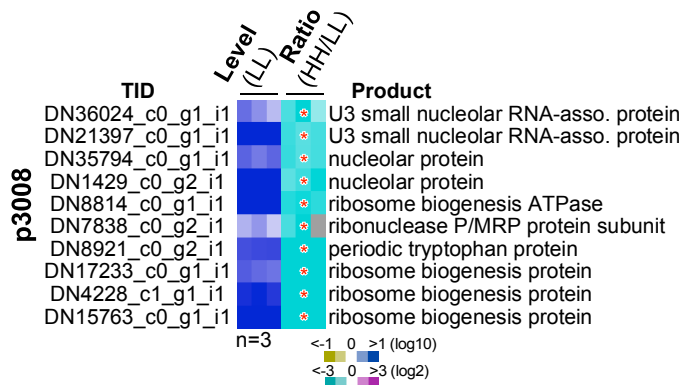

Supplement: Supplementary file 4 [file Data_Sheet_4.PDF]
