# Supplementary material for: Transcriptome and Metabolome Profiling of a Novel Isolate Chlorella sorokiniana G32 (Chlorophyta) Displaying Enhanced Starch Accumulation at High Growth Rate Under Mixotrophic Condition
Source: Front Microbiol. 2022 Jan 6;12:760307. doi: 10.3389/fmicb.2021.760307 (PMC8770532; doi:10.3389/fmicb.2021.760307)

Supplementary Figure S5. Comparison of maximum cell density, productivity, and content of energy-rich molecules between mixotrophic growth of *C. sorokiniana* M209220, UTEX1602, and G32 strains. (A) Growth curves (time vs DW) in this comparison were adapted from Figure 1C in Wan et al. 2011, Figure 1A in Li et al. 2014, and Figure 2A in this study. Four consecutive time points encompassing the maximum growth rate were selected from the indicated figures. Conversion of logarithmic form of DW is shown as Ln(DW) in table. (B) Growth curves of G32 in 2·BB and 3·BB media. Unit of light intensity and glucose supply is identical to (A).

A

Light intensity (μ mol m^{-2} s^{-1})	CO_2 supply	Glc supply (g L^{-1})	Time (d)	DW (g L^{-1})	Ln(DW)	Growth Rate (μ, d^{-1})	Productivity (g L^{-1} d^{-1})	Energy-rich molecule (%)	Reference (strain)
~200 (or 20K Lx)	Air	10	3	0.32	-1.139			50 (lipid) - (starch)	Wan et al., 2011 (M209220)
			4	0.52	-0.654	**0.486**	0.2		
			5	0.82	-0.198	0.455	0.3		
			6	**1.15**	0.140	0.338	**0.33**		
100	1% CO_2	4	1	0.8	-0.223			32 (lipid) - (starch)	Li et al., 2014 (UTEX1602)
			2	3	1.099	**1.322**	**2.2**		
			3	3	1.099	0	0		
			4	**3.1**	1.131	0.0328	0.1		
100	1% CO_2	6	1	0.8	-0.223			33 (lipid) - (starch)	Li et al., 2014 (UTEX1602)
			2	4.2	1.435	**1.658**	**3.4**		
			3	**4.5**	1.504	0.069	0.3		
			4	4.2	1.435	-0.069	-0.3		
50	Not supplied	5 (2BB)*	1	0.65	-0.431			26 (starch) 10 (lipid)	This study (G32)
			2	2.2	0.788	**1.219**	**1.55**		
			3	**2.5**	0.916	0.128	0.3		
			4	**2.5**	0.916	0	0		
100	Not supplied	6 (3BB)*	1	0.65	-0.431			28 (starch) 10 (lipid)	This study (G32)
			2	4.2	1.435	**1.866**	**3.55**		
			3	**4.5**	1.504	0.069	0.3		
			4	**4.5**	1.504	0	0		

Note: DW (g L^{-1}), maximum dry weight is shown in bold; Growth rate (d^{-1}), maximum growth rate is shown in bold; Productivity (g L^{-1} d^{-1}), maximum productivity is shown in bold. Energy-rich molecule (%), "-" stands for low content or no data. Asterisk (*) stands the strength of BB medium used in the study.

B

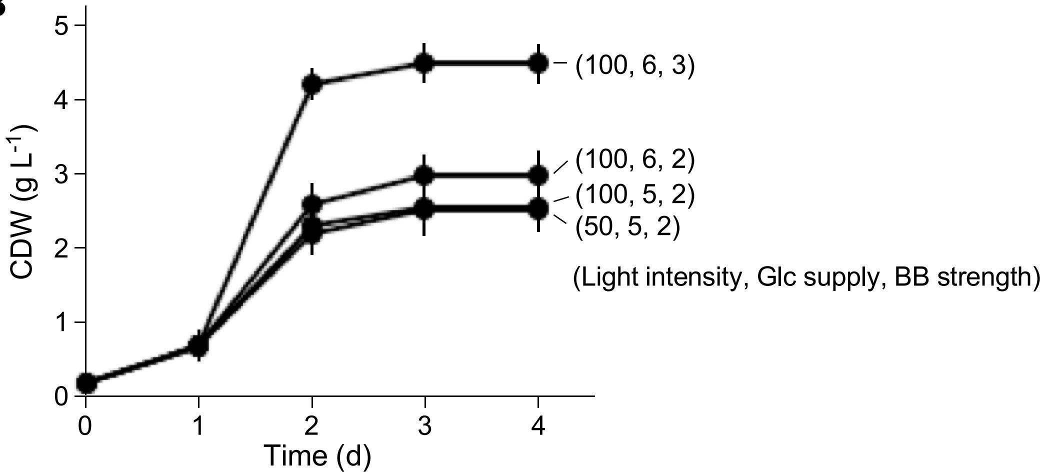

Supplement: Supplementary file 5 [file Data_Sheet_5.PDF]
