# Supplementary material for: Transcriptome and Metabolome Profiling of a Novel Isolate Chlorella sorokiniana G32 (Chlorophyta) Displaying Enhanced Starch Accumulation at High Growth Rate Under Mixotrophic Condition
Source: Front Microbiol. 2022 Jan 6;12:760307. doi: 10.3389/fmicb.2021.760307 (PMC8770532; doi:10.3389/fmicb.2021.760307)

Supplementary Figure S6. Transcription profiles of enzymes EC 1.2.1.12 (GAPDH, glyceraldehyde 3-phosphate dehydrogenase), EC 2.7.9.1 (PPDK, pyruvate orthophosphate dikinase), EC 2.6.1.1 (AspAT, aspartate aminotransferase), and EC 4.1.1.31 (PPC, phosphoenolpyruvate carboxylase).

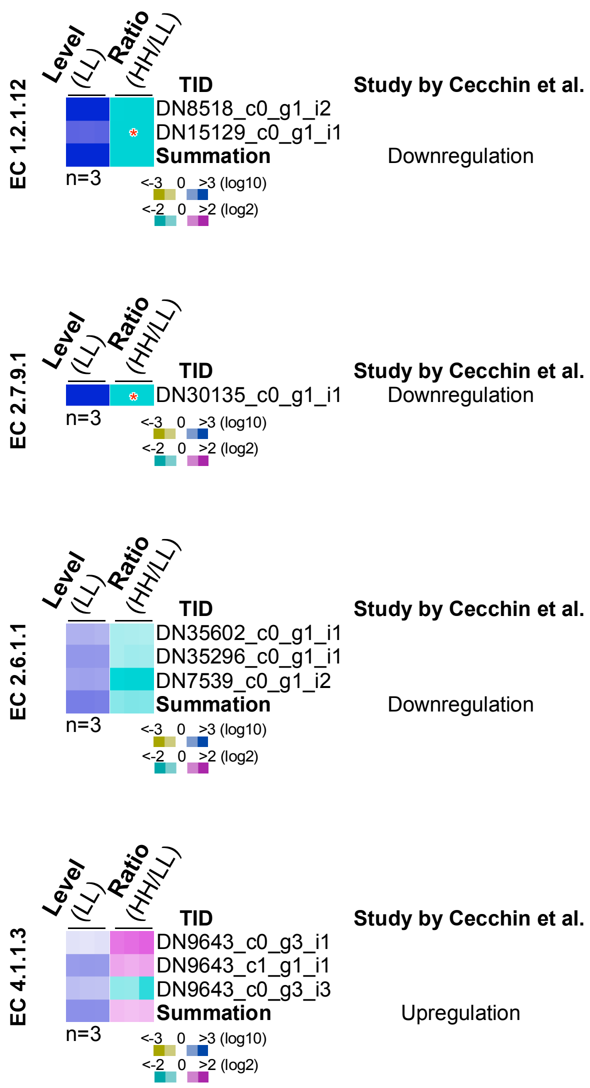

Supplement: Supplementary file 6 [file Data_Sheet_6.PDF]
